# Optimizing the Method for Differentiation of Macrophages from Human Induced Pluripotent Stem Cells

**DOI:** 10.1155/2022/6593403

**Published:** 2022-03-03

**Authors:** Shanshan Li, Lili Song, Yingwen Zhang, Zhiyan Zhan, Yi Yang, Lisha Yu, Hua Zhu, Weihua Huang, Wanqiao Wang, Haizhong Feng, Yanxin Li

**Affiliations:** ^1^Pediatric Translational Medicine Institute, Shanghai Children's Medical Center, School of Medicine, Shanghai Jiao Tong University, National Health Committee Key Laboratory of Pediatric Hematology & Oncology, Shanghai 200127, China; ^2^Department of Hematology & Oncology, Shanghai Children's Medical Center, School of Medicine, Shanghai Jiao Tong University, National Health Committee Key Laboratory of Pediatric Hematology & Oncology, Shanghai 200127, China; ^3^Department of Transfusion Medicine, The First Affiliated Hospital of Naval Medical University, Shanghai 200433, China; ^4^State Key Laboratory of Oncogenes and Related Genes, Renji-Med X Clinical Stem Cell Research Center, Renji Hospital, Shanghai Cancer Institute, Shanghai Jiao Tong University School of Medicine, Shanghai 200127, China

## Abstract

Macrophage is a very promising cell type for cancer immunotherapy, yet it is difficult to obtain enough functional macrophages for clinical cell therapy. Herein, we descibe a reliable method to produce functional macrophages through the differentiation of human induced pluripotent stem cells (hiPSCs). By optimizing the size control of embryoid bodies (EBs), we accelerated the differentiation process of macrophages and increased the production of macrophages without attenuating macrophage functions. Our final yield of macrophages was close to 50-fold of starting iPSCs. The macrophages showed phagocytic capacity in vitro and a xenograft tumor model. M0 macrophages could be further polarized into M1 and M2 subtypes, and M1 cells exhibited typical proinflammatory characteristics. Moreover, we found that hematopoietic differentiation originated from the outside of EB and matured inward gradually. Taken together, our protocol provides an effective method for the generation of macrophages comparable to blood-derived macrophages, which provides potential value for cell therapy and gene editing studies.

## 1. Introduction

Adoptive cell therapy, such as chimeric antigen receptor (CAR) T cell therapy, provides a revolutionary approach for cancer therapy, but its application in solid tumors still has some limitations [1]. Macrophages, the most plastic type of white blood cells of the immune system, play important roles in development, homeostasis, and cancer [2]. Since they can immerse in the tumor environment, enhance the cytotoxic effects of T cells, and have less no-tumor toxicity, CAR-macrophage has shown more advantages in the treatment of solid tumors [3]. However, due to the characteristic of terminal differentiation of macrophages [4], it is hard to obtain adequate amounts of cells.

The most common sources of human macrophages include peripheral blood or bone marrow monocytes, myeloid cell lines (such as THP-1), and pluripotent stem cells. Nevertheless, isolation of monocytes is time-consuming and the yield is low, and the myeloid cell lines cannot replicate the complexity of macrophages in vivo [5]. Macrophages derived from human embryonic stem cells have the same functions as macrophages from peripheral blood and bone marrow monocytes, but there are ethical issues involved [5]. hiPSCs become a favorable choice, because they have no ethical limitations and can be easily manipulated by genome editing technology [6–9].

In the past few years, the differentiation schemes of iPSC-derived macrophages (IPSDMs) have been constantly inproved and used in disease models [10–15], macrophage-pathogen interaction modeling [16, 17], and cell therapy [18]. Initially, cells in some methods were required to coculture with OP9 mouse stromal cells [10, 19, 20], but this approach was gradually obsoleted because of the use of xenogeneic cells that limited the clinical application. At present, hiPSC differentiation schemes are mainly based on the form of EB or monolayer cultivation (Table S1). Compared with monolayer cultivation protocols, EB protocols have the advantage of allowing a continuous harvest of suspension cells for the long-term production of macrophages with relatively few cytokines [21–26]. By contrast, monolayer cultivation protocols achieved higher one-off collection rates of macrophage by modified differentiation steps, such as a complex mixture of factors [27–30] (up to 11 factors), hypoxia conditions [28], and CD14 sorting [29, 30]. In the past, the seeding number of iPSCs per EB in most EB-based protocols was unfixed. The size of EB was random and the yield was not stable [22–25, 31, 32]. Continuous generation of IPSDMs resulted in high cumulative yield, but the efficiency of amplification was not high enough to obtain sufficient quantities of macrophages in a short time [21, 22, 25]. Thus, it is necessary to establish a convenient and less expensive differentiation scheme that can achieve higher yields in the short term.

In this study, by optimizing EB size, we accelerated the differentiation process of macrophages and increased the production of macrophages without attenuating macrophage functions.

## 2. Material and Methods

### 2.1. hiPSC Culture

hiPSCs were cultured in feeder-free, serum-free PSCeasy® II medium (Cellapy) and were passaged when the cells reached 70%-80% confluency. To passage cells, the medium was aspirated and 1 ml 0.5 mM EDTA (Invitrogen) was added to each well of one 6-well plate, and then, cells were incubated for 4-5 min at 37°C. Each well of one 6-well plate could be transferred to one 6-well plate by 1 : 6 split ratio. 2 ml medium per well was dispensed to Matrigel (BD Biosciences)-coated 6-well plates, and the plate was returned to an incubator at 37°C, 5% CO_2_.

### 2.2. Differentiation of hiPSCs into Macrophages

We performed hematopoietic differentiation of hiPSCs by using two versions of the EB-based hematopoietic differentiation protocol. First, hiPSC colonies were incubated with 1 ml TrypLE Express (Gibco) for 2 min at room temperature. In the P3500 protocol, the TrypLE Express was aspirated, and the cells were resuspended with APEL (STEMCELL Technologies) and disrupted to a single cell suspension by pipetting up and down, supplemented with 10 *μ*M Rock inhibitor Y27632 (STEMCELL Technologies), 10 ng/ml BMP4 (R&D Systems), and 10 ng/ml recombinant human bFGF (R&D Systems). Cells (3500/well) were seeded into 60 wells in the middle of an untreated round-bottom 96-well plate (Costar 3788) and centrifuged at 300 × *g* at room temperature for 5 min. From day 2 to day 14, the cells were cultured in APEL containing BMP4 (10 ng/ml), bFGF (10 ng/ml), VEGF (PeproTech) (10 ng/ml), and SCF (PeproTech) (50 ng/ml). On day 14, the APEL medium was removed, and then, macrophage differentiation medium RPMI Medium 1640 basic (Gibco) was added, supplemented with 10% fetal bovine serum (FBS, Gibco), 1% penicillin-streptomycin (PS, NCMBIO), 100 ng/ml M-CSF (PeproTech), and 25 ng/ml IL-3 (PeproTech). On day 22, both EBs and single cells in suspension were harvested and then filtered through 100 *μ*m cell strainers to remove the EBs. The cells were centrifuged at 1000 rpm for 5 min and seeded into the 6-well tissue culture plate. After day 22, aspirate the spent medium and add fresh macrophage differentiation medium (RPMI 1640+10% FBS+1% PS+100 ng/ml M-CSF) as needed if the medium turned yellow.

In the P8000 protocol, the cells were resuspended with APEL supplemented with 10 *μ*M Rock inhibitor Y27632, 10 ng/ml recombinant human bFGF, 20 ng/ml BMP4, 20 ng/ml VEGF, and 40 ng/ml SCF. Cells (8000/well) were seeded into 60 wells in the middle of nontreated round-bottom 96-well plates in 100 *μ*l medium per well. From day 0 to day 8, 100 *μ*l APEL medium was removed and the above medium (no Y27632) was added every 2~3 days. On day 8, aspirate APEL medium and replace with fresh macrophage differentiation medium (RPMI1640+10% FBS+1% PS+100 ng/ml M-CSF+25 ng/ml IL-3). On day 14, the EBs and cells were collected and seeded into round-bottom 6-well plates (Costar 3471), and suspension cells were harvested in the supernatants. The EBs were removed until day 22. The rest was as described in the P3500 protocol.

### 2.3. Flow Cytometry Analysis

Cells were washed once and stained with antibodies or isotype control for 30 minutes at 4°C in flow cytometry buffer. The samples were analyzed on FACS Calibur (BD Canto Plus), and data were analyzed using FlowJo v10 (FlowJo, LLC). The following antibodies were used: CD45-APC-Cy7 (2D1, IgG1*κ*-APC-Cy7) from BioLegend; CD34-PE (4H11, IgG1*κ*-PE) and CD14-PE (61D3, IgG1*κ*-PE) from Invitrogen; CD11b-APC (M1/70, IgG2b*κ*-APC), CD80-PE (2D10.4, IgG1*κ*-PE), CD86-PE-Cy7 (IT2.2, IgG2b*κ*-PE-Cy7), CD163-PE (GHI/61, IgG1*κ*-PE), and CD206-PE-Cy7 (19.2, IgG1*κ*- PE-Cy7) from eBioscience.

### 2.4. Wright-Giemsa Staining

Cells were centrifuged at 500 rpm for 5 minutes and immobilized on microscope slides using cytospins (Tharmac Cellspin) followed by staining with Wright-Giemsa stain.

### 2.5. CD14^+^ Monocyte Isolation

Peripheral blood mononuclear cells (PBMCs) were isolated from healthy donor blood by Ficoll-Paque (Cytiva), and CD14^+^ monocytes were isolated from PBMCs using human CD14 Microbeads (Miltenyi Biotec) according to the manufacturer's instructions.

### 2.6. Differentiation of Macrophage Subtypes

The cells were differentiated into M0 cells at day 25 to day 30. M0 cells were polarized for 48 h to M1 cells in macrophage differentiation medium supplemented with 100 ng/ml LPS (Sigma) and 20 ng/ml IFN-*γ* (PeproTech), to M2 cells in macrophage differentiation medium supplemented with 20 ng/ml IL-4 (PeproTech). Cells were detached using Accutase (Gibco) or 5 mM EDTA for 5-10 minutes.

### 2.7. Fluorescent Bead Phagocytosis Assay

Fluoresbrite™ Polychromatic Red 1.0 *μ*m Latex beads (Polyscience Inc., 18660) were added to the cell culture medium. After 24 hours, macrophages were washed and analyzed by confocal microscopy or flow cytometry.

### 2.8. Tumor Phagocytosis Assay

Reh and Nalm6 tumor cells were stained with Hoechst 33342 (Life Technologies) at 37°C for 20 minutes. Then, the cells were washed with PBS three times and 1 × 10^5^ macrophages were cocultured with tumor cells in the proportions of 1 : 1, 1 : 2, or 1 : 4, respectively, and then incubated for 2 hours at 37°C. Cells were seeded into a round-bottom 96-well plate at a density of 1 × 10^5^/well in 200 *μ*l macrophage medium. The cells were harvested and stained with CD11b-APC antibody at 4°C for 30 minutes, and then, the phagocytosis ratio was measured by FACS Calibur. Macrophages were cocultured with tumor cells in the proportions of 1 : 4 in M0, M1, and M2 phagocytosis assay.

### 2.9. Immunofluorescence

First, cells were fixed in 4% paraformaldehyde (PFA) for 20 minutes and then washed three times using PBS. For bead phagocytosis assay, the cells were stained with 5 *μ*g/ml Wheat Germ Agglutinin (WGA), Alexa Fluor 633 Conjugates (Invitrogen) for 10 minutes and then washed twice using PBS followed by DAPI (1 : 500) staining as standard procedures. Undifferentiated hiPSCs were used as control. For the tumor phagocytosis assay, the cocultured cells were blocked with 10% goat serum and then incubated with CD11b-APC antibody at 4°C overnight; then, the antibody was washed away. The images were captured by a confocal laser scanning microscope (Leica SP8).

### 2.10. Frozen Section and Immunofluorescence of EBs

EBs were collected and embedded using optimal cutting temperature compound followed by frozen section, and the microscope slides were stored at -80°C. For immunofluorescence, microscope slides were left at room temperature for 15 minutes and immersed in PBS for 10 minutes to remove OCT. Then, EBs were blocked with 10% goat serum and incubated with CD34-PE and CD45-APC-Cy7 antibody (1 : 100) at 4°C overnight, and then, the cells were washed and stained with DAPI as described above.

### 2.11. Cytokine Detection

M0 macrophages were cultured in macrophage differentiation medium until reaching more than 90% confluency. Then, M0 cells were polarized toward M1 and M2 cells with previously mentioned cytokines. Cell supernatants were collected and the concentration of cytokines was detected by AimPlex® multiplex immunoassays for Flow™ (QuantoBio).

### 2.12. Animal Study

All mouse studies were conducted in accordance with national guidelines for the humane treatment of animals and were approved by the Institutional Animal Care and Use Committee (IACUC) at Shanghai Jiao Tong University. CB17-SCID mice were divided into three groups, and cells were resuspended in 100 *μ*l matrix and subcutaneously inoculated into the mice as (a) 2 × 10^6^ Raji cells each mouse, (b) 2 × 10^6^ Raji cells plus 7 × 10^6^ PBDMs each mouse, and (c) 2 × 10^6^ Raji cells plus 7 × 10^6^ IPSDMs each mouse. Tumor formation was observed closely, and tumor volume was measured every 2-3 days when the tumors were visible. When the tumor grew to a certain size, the mice were sacrificed and tumors were dissected, photographed, and weighed. Tumor tissue was embedded in paraffin blocks for sectioning and staining with hematoxylin and eosin.

### 2.13. Statistical Analysis

All experiments were repeated three times or more. Two-tailed Student's unpaired *t*-test was used for pairwise statistical analysis, and multiple groups were evaluated by one-way ANOVA or two-way ANOVA followed by Bonferroni's multiple comparisons test. Data are presented as the mean ± SD. *P* < 0.05 was considered statistically significant.

## 3. Results

### 3.1. The Quantity of Seeding Cells Affected the Process of hiPSCs Differentiated into Macrophages

**Figure 1:**
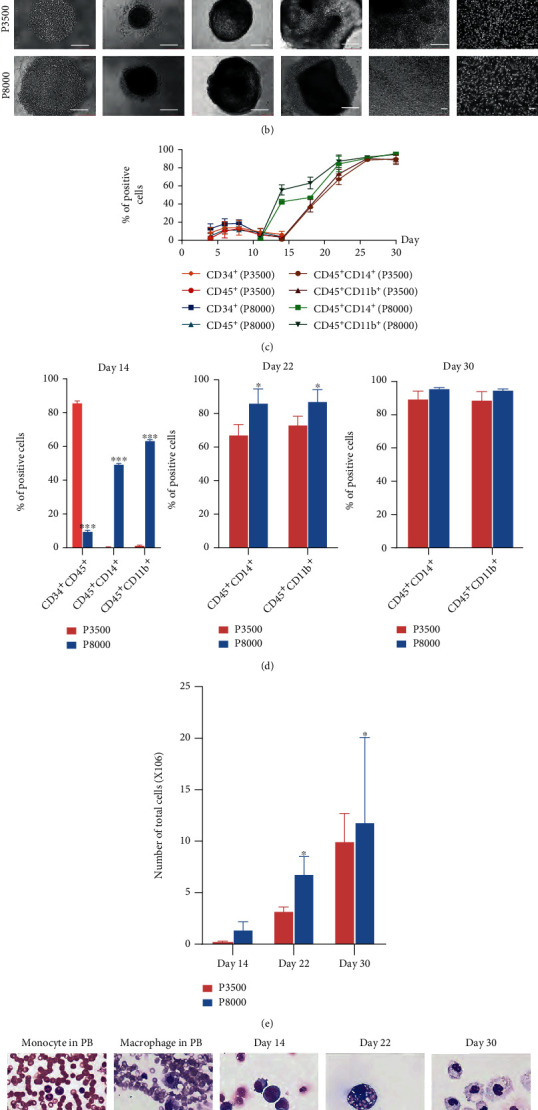
The quantity of seeding cells affected the process of hiPSCs differentiated into macrophages. (a) Schematic diagram of the protocols of macrophage differentiation from hiPSCs. (b) Representative bright-field images of EBs and cells at different time points during culture stages under two macrophage differentiation protocols. Scale bars, 250 *μ*m. (c) The percentages of different cell types (CD34^+^, CD45^+^, CD45^+^CD14^+^, and CD45^+^CD11b^+^) produced during the different culture stages under two macrophage differentiation protocols. (d) The percentage of CD34^+^CD45^+^ cells at day 14 and CD45^+^CD14^+^/CD45^+^CD11b^+^ cells at day 14, day 22, and day 30 in two macrophage differentiation protocols. ^∗^*P* < 0.05 and ^∗∗∗^*P* < 0.001. (e) The total number of cells at day 14, day 22, and day 30 in two macrophage differentiation protocols. ^∗^*P* < 0.05. (f) Representative Wright-Giemsa staining images of monocytes and macrophages in peripheral blood and cells differentiated from hiPSCs at day 14, day 22, and day 30 in the P3500 protocol. Scale bar represents 50 *μ*m.

Macrophage differentiation from iPSCs needs three steps: hematopoietic differentiation, expansion of myeloid progenitors, and maturation of macrophages. Here, we used two protocols, P3500 and P8000, to compare macrophage differentiation efficacy from hiPSCs (Figure 1(a)). In the P3500 protocol, hiPSCs (3500/well) were seeded in an untreated round-bottom 96-well plate and the culture medium was replaced with macrophage differentiation medium on day 14. In the P8000 protocol, hiPSCs (8000/well) were seeded and the culture medium was replaced on day 8. The percentage of CD34^+^CD45^+^ cells was more than 80% on day 14 in the P3500 protocol (Figure 1(d), Figure S1a), but the rate of CD34^+^CD45^+^ cells was no more than 20% and the CD45^+^CD14^+^/CD45^+^CD11b^+^ cells have reached 40%~60% at day 14 in the P8000 protocol (Figures 1(c) and 1(d), Figure S1a). Myeloid cells could be harvested from the EB culture supernatant in the P8000 protocol at day 14, which was earlier than that in the P3500 protocol (Figures 1(b)–1(d)). From day 14 to day 22, CD34^+^CD45^+^ hematopoietic progenitor cells (HPCs) were differentiated into myeloid progenitor cells in the P3500 protocol. On day 22, the percentage of CD45^+^CD14^+^/CD45^+^CD11b^+^ cells reached about 70% in the P3500 protocol, while that positive rate of P8000 protocol was higher (Figures 1(c) and 1(d), Figure S1b). At the end of the second step, cells can be frozen or differentiated into a more mature state. On day 30, there were no differences in the percentage of CD45^+^CD14^+^/CD45^+^CD11b^+^ cells between the two protocols (Figures 1(c) and 1(d), Figure S1c). Overall, the differentiation process of macrophages in the P8000 protocol was faster than that in the P3500 protocol. More importantly, the number of total cells harvested in the P8000 protocol was higher than that of the P3500 protocol on day 22 and day 30 (Figure 1(e)). At last, about (1 ~ 2) × 10^7^ cells were collected per 96-well plate in the P8000 protocol at day 30. During the differentiation process of the P3500 protocol, the cells of day 14, day 22, and day 30 were stained by Wright-Giemsa stain (Figure 1(f)). On day 14, there were mainly immature hematopoietic cells. On day 22, the monocyte showed typical morphological characteristics and had more vacuoles in the cytoplasm than that of monocytes in peripheral blood. On day 30, the cells were more mature, like “fried eggs.” These results suggested that the quantity of seeding cells and time to add cytokines determined the differentiation efficiency.

### 3.2. More Seeding Cells Promoted the Development of HPCs in EBs

**Figure 2:**
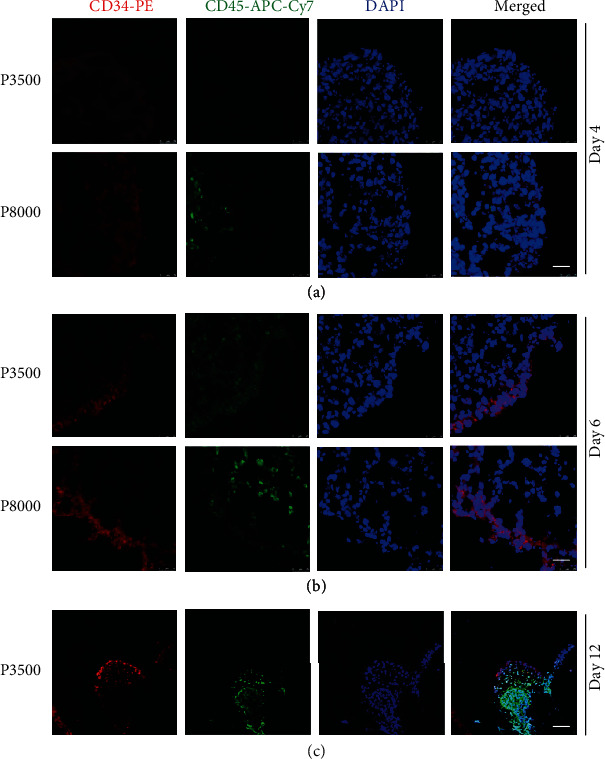
More seeding cells promoted the development of HPCs in EBs. (a–c) Frozen sections and immunofluorescence images of EBs in two macrophage differentiation protocols. Immunocytochemical analysis of CD34 (red) and CD45 (green) at day 4, day 6, and day 12. DAPI (blue) shows the cell nuclei (scale bars: 25 *μ*m in (a) and (b), 50 *μ*m in (c)).

To further explore the development of HPCs in EBs, EBs of the two protocols at different time points were observed by freezing section and immunofluorescence staining. On day 4, CD34^+^ and CD45^+^ cells could be observed in the P8000 protocol, but hardly in the P3500 protocol by immunofluorescence (Figure 2(a)). On day 6, CD34^+^ and CD45^+^ cells were first found in the P3500 protocol by immunofluorescence (Figure 2(b)). In addition, we found that CD34 protein was mainly located in the outer layer of EBs which directly interacted with cytokines and gradually decreased as it went inward, while CD45 was mainly expressed in the inner layer of EBs which was going weaker outward (Figures 2(a)–2(c)). These results verified that HPCs were differentiated from EB outer layer to internal sac and the bigger EB promoted HPC differentiation.

### 3.3. The hiPSC-Derived Macrophages Possess the Function of Phagocytosis

**Figure 3:**
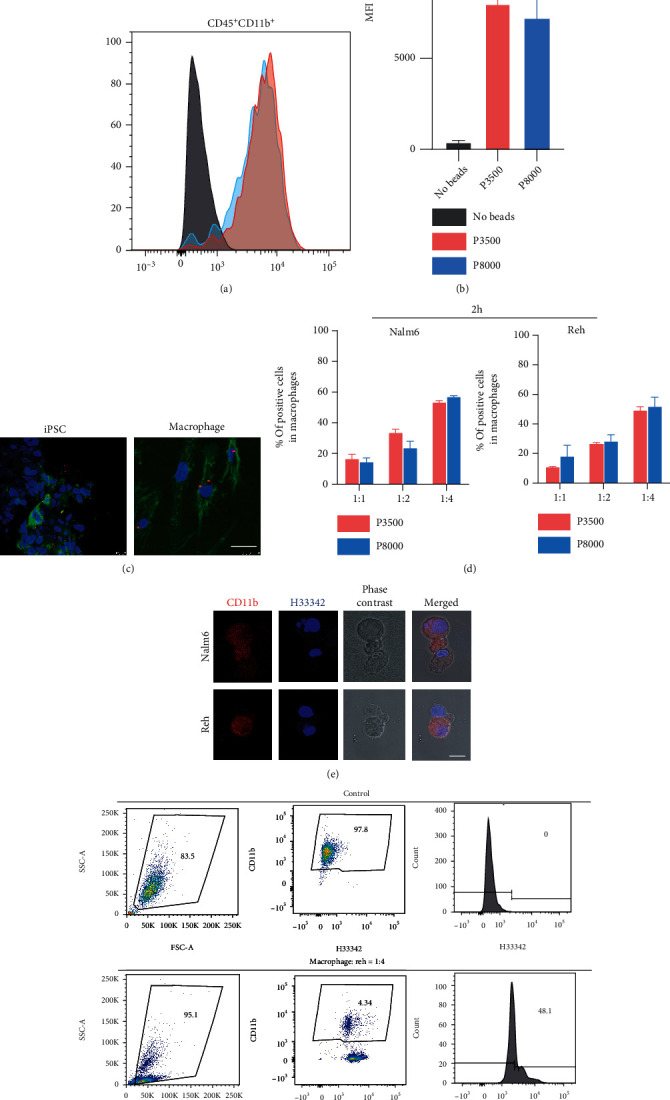
The hiPSC-derived macrophages possess the function of phagocytosis. (a, b) Flow cytometric analysis of the uptake of fluorescent beads by IPSDMs in two differentiation protocols. (c) Phagocytosis of fluorescent beads (red) by IPSDMs and IPSCs control. DAPI (blue) shows the cell nuclei; WGA (green) shows the cell membrane. Scale bars, 50 *μ*m. (d) When the ratio of IPSDMs to tumor cells is 1 : 1, 1 : 2, or 1 : 4, the proportion of IPSDMs that have engulfed tumor cells is calculated. All experiments were performed three times. Error bars, SD. (e) Representative images of Nalm6 and Reh cells (labeled with Hoechst 33342) phagocytized by IPSDMs (labeled with CD11b and phase contrast image). Scale bars, 25 *μ*m. (f) Flow cytometric analysis of phagocytosis of IPSDMs on Reh (IPSDM : Reh = 1 : 4). CD11b^+^ macrophages are gated (middle panel), and V450 intensity is shown as a histogram (right panel). Macrophages cocultured with Reh-Hoechst 33342 suspension (after washing) as a negative control.

Peripheral blood-derived macrophages (PBDMs) can phagocytose foreign substances, so we wanted to see if macrophages derived from hiPSCs also have phagocytic function. Firstly, we used the fluorescent beads to determine the phagocytic function of macrophages as previously described [28]. After macrophages were incubated with fluorescent beads for 24 hours, the macrophages were observed with laser confocal microscopy. To exclude a small number of fluorescence beads still stuck to the cells causing false positives, we used hiPSCs as a control. Obviously, the fluorescent beads were located inside the macrophages, while the fluorescent beads were scattered on the surface in the hiPSCs group (Figure 3(c)). Flow cytometry also confirmed the phagocytosis of fluorescent beads by macrophages, and there was no difference in the phagocytosis of macrophages between the two differentiation protocols (Figures 3(a) and 3(b)).

Next, we further verified the phagocytosis ability of macrophages toward tumor cells. First, leukemia cell lines, Nalm6 and Reh, were labeled with Hoechst 33342 fluorochrome, and then, macrophages and tumor cells were coincubated. When the ratio of macrophages to tumor cells was 1 : 4, the proportion of macrophages that had phagocytosed tumor cells was the highest, which was above 40%-50% (Figures 3(d) and 3(f)). We hypothesized that increased contact between tumor cells and macrophages promoted the phagocytosis of macrophages. The phagocytosis of macrophages on tumor cells was also captured by laser confocal microscopy (Figure 3(e)). The dynamic process of phagocytosis was recorded by real-time fluorescence imaging of living cells (Supplementary Data, Movie).

To determine whether differentiated macrophages also have antitumor activity in vivo, Raji cells with or without PBDMs or IPSDMs were subcutaneously inoculated into CB17-SCID mice. After more than one month of observation, there were no significant differences in the tumor weight and size among the three groups (Figures S2a and S2b). However, in the group treated with PBDMs or IPSDMs, dissolution and cavities were found inside the tumor tissues (Figure S2c). These results suggested that although our IPSDMs did not show an obvious antitumor effect in vivo, the function of IPSDMs in our protocol was comparable to that of PBDMs.

### 3.4. Identification of the Different Polarization Subtypes from IPSDMs

**Figure 4:**
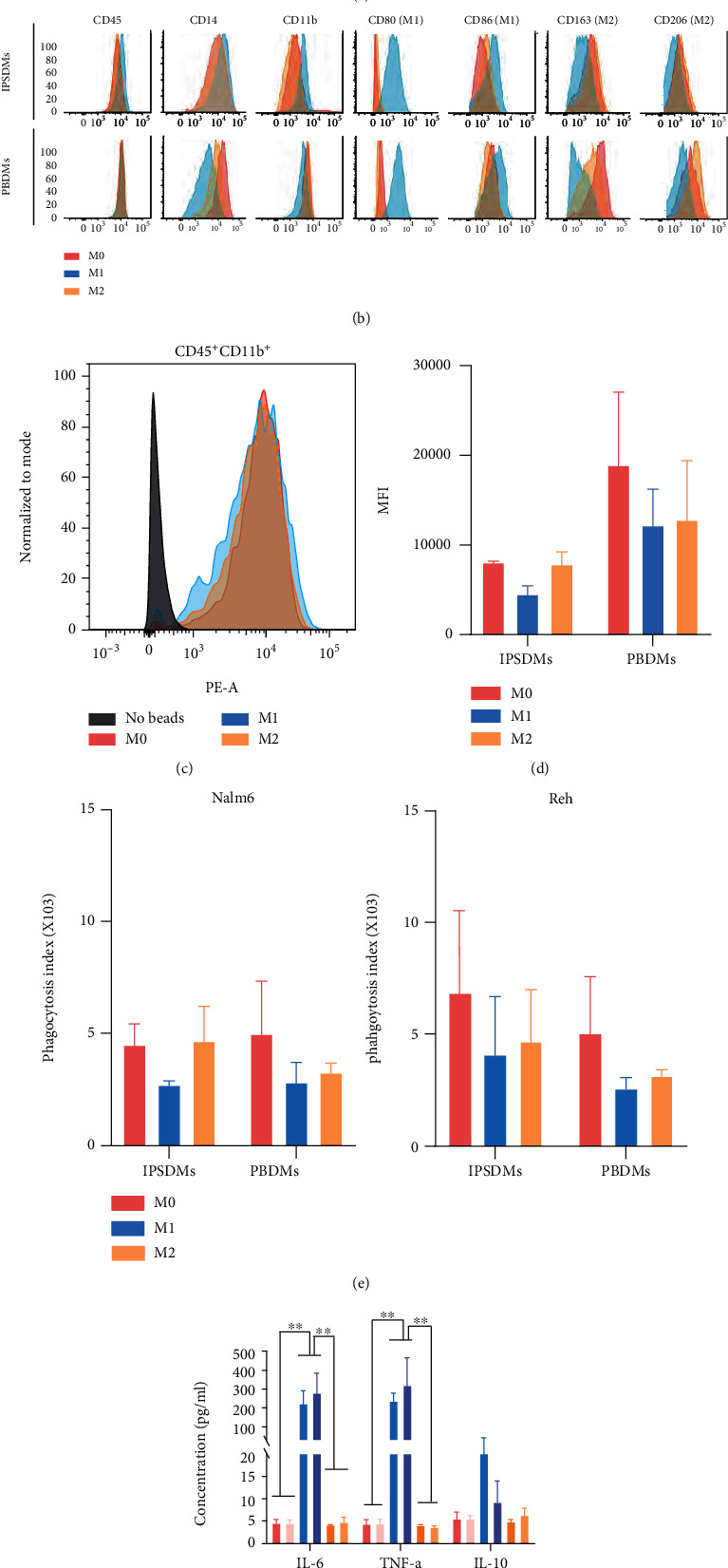
Identification of the different polarized subtypes from IPSDM. (a) Representative bright-filed images of M0 (left panel), M1 (middle panel), and M2 cells (right panel) from hiPSCs. Scale bars, 100 *μ*m. (b) Flow cytometric analysis of markers CD45, CD14, CD11b, CD80 (M1), CD86 (M1), CD163 (M2), and CD206 (M2) of IPSDMs and PBDMs. (c, d) Flow cytometric analysis of the uptake of fluorescent beads by subtypes of IPSDMs and PBDMs. All experiments were performed three times. Error bars, SD. (e) Flow cytometric analysis of phagocytosis of IPSDM and PBDM subtypes on Nalm6 (labeled with Hoechst 33342) and Reh (labeled with Hoechst 33342) (IPSDMs : tumor cells = 1 : 4). Phagocytosis index: the percentage of V450^+^ macrophages multiplied by the MFI of V450. Error bars, SD. (f) Concentration of IL-6, IL-10, and TNF-*α* in the supernatants of M0, M1, and M2 cells after 48-hour polarization. ^∗∗^*P* < 0.01.

Macrophages are usually divided into two different activated states, namely, M1 (or classical activated) type, and M2 (or alternately activated) type. M1 phenotype is proinflammatory which has strong antimicrobial and antitumor activity, while M2 is considered to promote tissue remodeling and tumor growth [33]. Under continuous induction of M-CSF, the myeloid progenitor cells adhered to the six-well plate and were differentiated into M0 macrophages. M0 cells could be polarized toward M1 cells by LPS and IFN-*γ*. Using IL-4, M0 cells could be polarized toward M2 cells. M0 cells were elongated in shape, M1 had more protrusions, and M2 cells were more rounded (Figure 4(a)), as previously described [29]. The expression of CD80 and CD86 was upregulated in M1 cells, and the expression of CD163 and CD206 was higher in M0 and M2 cells (Figure 4(b)). PBDMs and IPSDMs obtained similar results. Furthermore, the minor difference between IPS-M0 and IPS-M2 cells showed that the phenotype of M-CSF-induced macrophages had shifted toward M2 cells, as discussed by Zhang et al. [31]. Then, we investigated whether the phagocytic capacity of macrophages in different activated states differed. The results demonstrated that there was no difference in the phagocytic capacity between M0 and M2 cells, while that of M1 cells slightly decreased but had no statistical significance (Figures 4(c)–4(e)). And more, the phagocytic activity of IPSDMs was comparable to that of PBDMs (Figures 4(d) and 4(e)). Cytokine secretion showed that significantly higher levels of proinflammatory cytokines (IL-6 and TNF-*α*) were secreted in PB-M1 and IPS-M1 cells (Figure 4(f)). The results confirmed that macrophages from iPSCs could be polarized into different subtypes as macrophages derived from PB.

## 4. Discussion

In this study, we show an optimized differentiation method of IPSDMs to produce mature macrophages in a relatively short time with a higher yield. Macrophages have always been a hot topic in cancer research due to its close relationship with tumors, and autologous macrophages have been used in clinical trials for cancer treatment in the last century [34]. Recently, CAR-macrophages have been shown to induce a proinflammatory tumor microenvironment and boost antitumor T cell activity in humanized mouse models [3, 35]. Thus, our protocol will facilitate the research and subsequent clinical application of IPSDMs.

In this study, we adjusted the size of EB by increasing the seeding number of iPSCs and produced mature macrophages in a relatively short time with a higher yield. Our final yield of macrophages was close to 50-fold of starting iPSCs, about ~2 × 10^7^ macrophages per 96-well plate in a month. Compared to the method of van Wilgenburg et al. [21], the yield of macrophages in our protocol was increased by 10-fold in the first month of differentiation (Table S1). According to the seeding iPSCs, the amplification efficiency of our protocol was higher than that of most protocols by one month (Table S1). Recently, Gutbier et al. modified the protocol of van Wilgenburg et al. [21], and the calculated yield per iPSC (by one month) was comparable to that of our protocol (Table S1) [36]. Di Cui et al. developed a high-yield monolayer differentiation protocol for about 2 × 10^4^ monocytes per seeded hiPSC [30]. The significantly larger scale in cell production and efficient cryopreservation partially compensated for the other limitations of monolayer cultivation. For our research, how to further expand the cumulative yield and extend duration will be the focus. Considering the diversity of various protocols, including PSC lines, culture conditions, differentiation time, and cost performance, the future direction might be to identify the cost-effective, standardized protocols for large-scale clinical application.

Here, we revealed that hematopoietic differentiation originated from the outside of EB and matured inward gradually. The initial size of EB determined the differentiation efficiency but did not affect the phagocytosis of macrophages. Although the most widely known theory is that macrophages derived from circulating monocyte originate in the bone marrow, studies in recent years have shown that some tissue-resident macrophages arise during early embryonic development, independently maintained in a steady state [37–40]. EB is structurally similar to the early stages of embryonic development, which can simulate the original hematopoietic process from extraembryonic structure yolk sac in mammals.

Although our IPSDMs did not show an obvious antitumor effect in vivo, the function of IPSDMs in our protocol was comparable to that of PBDMs. Like PBDMs, our IPSDM treatment caused dissolution and cavities inside the tumor tissues. Compared to IPSDMs, Zhang et al. found that iPSC-derived, CAR-expressing macrophage cell- (CAR-iMac-) treated animals showed reduced ovarian tumor burden [18]. Klichinsky et al. also demonstrated that CAR-M-treated mice demonstrated a marked reduction in SKOV3 tumor burden [35]. Moreover, although all animals eventually progressed, a single infusion of CAR-Ms led to a prolongation of overall survival [35]. Thus, these demonstrate that IPSDMs in combination with CARs will exert its unique effects in clinical solid tumor treatment.

## 5. Conclusion

In summary, we describe an optimized and reproducible differentiation method to produce mature macrophages from iPSCs in a relatively short time with a higher yield, which may promote cell therapy of macrophages.

## Data Availability

Please contact author for data requests.

## Supplementary Materials

Supplementary Materials Figure S1: flow cytometric analysis of cells on day 14, day 22, and day 30. (a) The percentages of different markers (CD34^+^CD45^+^, CD45^+^CD14^+^, and CD45^+^CD11b^+^) of P3500 protocol (upper panel) and P8000 protocol (lower panel) on day 14. (b) The percentages of different markers (CD45^+^CD14^+^ and CD45^+^CD11b^+^) of P3500 protocol (upper panel) and P8000 protocol (lower panel) on day 22. (c) The percentages of different markers (CD45^+^CD14^+^ and CD45^+^CD11b^+^) of P3500 protocol (upper panel) and P8000 protocol (lower panel) on day 30. Figure S2: validation of the antitumor effect of macrophages in vivo. (a) A line chart of tumor volume over time and (b) the weight of tumors after the mice were sacrificed. (c) Hematoxylin-eosin staining of paraffin sections of tumor tissues. Arrows: macrophages. Scale bars, 250 *μ*m in the left column, 50 *μ*m in the middle and right column. Table S1: summary of protocols for differentiation of macrophages from iPSCs. A list of main publications for macrophage differentiation protocols including coculture with OP9 cells, EB-based protocols, and monolayer cultivation (tissue macrophage subsets such as microglia are not included). Movie: the phagocytosis of IPSDMs toward Reh-Hoechst 33342 was clearly observed in real-time fluorescence imaging of living cells (https://drive.google.com/file/d/1rLwt6xMU46n2u4J5JIOOqWWyHEeGCf7D/view?usp=sharing).
