# Supplementary material for: Optimizing the Method for Differentiation of Macrophages from Human Induced Pluripotent Stem Cells
Source: Stem Cells Int. 2022 Mar 3;2022:6593403. doi: 10.1155/2022/6593403 (PMC8913134; doi:10.1155/2022/6593403)
Supplement: Supplementary Materials — Figure S1: flow cytometric analysis of cells on day 14, day 22, and day 30. (a) The percentages of different markers (CD34+CD45+, CD45+CD14+, and CD45+CD11b+) of P3500 protocol (upper panel) and P8000 protocol (lower panel) on day 14. (b) The percentages of different markers (CD45+CD14+ and CD45+CD11b+) of P3500 protocol (upper panel) and P8000 protocol (lower panel) on day 22. (c) The percentages of different markers (CD45+CD14+ and CD45+CD11b+) of P3500 protocol (upper panel) and P8000 protocol (lower panel) on day 30. Figure S2: validation of the antitumor effect of macrophages in vivo. (a) A line chart of tumor volume over time and (b) the weight of tumors after the mice were sacrificed. (c) Hematoxylin-eosin staining of paraffin sections of tumor tissues. Arrows: macrophages. Scale bars, 250 μm in the left column, 50 μm in the middle and right column. Table S1: summary of protocols for differentiation of macrophages from iPSCs. A list of main publications for macrophage differentiation protocols including coculture with OP9 cells, EB-based protocols, and monolayer cultivation (tissue macrophage subsets such as microglia are not included). Movie: the phagocytosis of IPSDMs toward Reh-Hoechst 33342 was clearly observed in real-time fluorescence imaging of living cells (https://drive.google.com/file/d/1rLwt6xMU46n2u4J5JIOOqWWyHEeGCf7D/view?usp=sharing). [file 6593403.f1.zip › Table S1 (1).docx]

**Table S1**

| **Co-culture with OP9 cells** | | | | | | |
| --- | --- | --- | --- | --- | --- | --- |
| **Reference** | **Growth factors** | **Differentiation time** | **Yield (by one month)** | **Cumulative yield** | **phagocytosis** | **Special conditions** |
| Choi et al., 2011 | M-CSF, IL-1β | 16-18 days | ~1 cell/iPSC | NA | NA | Xenogeneic cells |
| Senju et al., 2011 | GM-CSF, M-CSF | 31-41 days | NA | NA | Zymosan; amyloid β; BALL-1 | Xenogeneic cells |
| Kambal et al., 2011 | GM-CSF, M-CSF | 13 days | NA | NA | NA | Xenogeneic cells |
| Brault et al., 2014 | GM-CSF, M-CSF | 28 days | NA | NA | Staphyloccocus aureus; Zymosan | Xenogeneic cells |

| **EB-based protocols** | | | | | | | | | | |
| --- | --- | --- | --- | --- | --- | --- | --- | --- | --- | --- |
| **Reference** | **Growth factors** | | **Myeloid cells harvest time** | **Duration** | **EB size** | **Calculated yield/cell (by one month)** | **Cumulative yield** | **Yield described in the article** | **Phagocytosis** | **Special conditions** |
| Van Wilgenburg  et al.,2013 | BMP4, VEGF, SCF, IL-3, M-CSF | | 14 days | 2 weeks-12 months | 1×10^4^ seeding iPSCs/EB | ~5 monocytes/iPSC | ~50 cells/iPSC by a year | ≥1×10^7^ PSC-MC per plate (6-well) after 8 weeks, up to ~3×10^7^ monocytes per plate (6-well) by a year | Zymosan | NA |
| Lachmann et al.,2015 | bFGF, IL-3, M-CSF | | 15-20 days | 15 days-5 months | Unfixed cell numbers/  EB | NA  (Without seeding data) | NA | (0.5-1) ×10^6^ cells/week/well per plate (6-well) for 2 months, up to 4-5 months | Latex beads | Orbital  shaker |
| Zhang et al.,2015 | BMP4, VEGF, SCF, bFGF, TPO, FLT3L, M-CSF | | 15 days | 22 days | Unfixed cell numbers/  EB | ~5 macrophages/  iPSC | 2 × 10^7^ macrophages/ plate (6-well) of iPSCs  (one-off collection) | Up to 2 × 10^7^ of CD45^+^/CD18^+^ differentiated  macrophages per plate (6-well) of confluent iPSCs for 22 days | Zymosan | Hypoxia |
| Ackermann et al., 2018 | IL-3, M-CSF | | 15-20 days | 15 days-7 weeks | Unfixed cell numbers/  EB | NA  (Without seeding data) | 250ml bioreactor: ~1.5×10^8^ cells | 250ml bioreactor: (1-3) × 10^7^ cells  From week 3, maintained for more than 5 weeks | pHrodo™ Red E. coli BioParticles; P.aeruginosa PAO1 | Orbital  shaker |
| Mukherjee et al.,2018 | IL-3, M-CSF | | 4 weeks | 4 weeks-8 months | Unfixed cell numbers/  EB | NA | NA | Myeloid precursors can be harvested every 4-5 days, continued for 6-8 months | NA | NA |
| Shi et al., 2019 | BMP4, VEGF, SCF, bFGF, TPO, FLT3L, M-CSF | | 15 days | 22 days | Unfixed cell numbers  /EB | ~5 macrophages /iPSC | 2 × 10^7^ cells /plate (6-well) of iPSCs  (one-off collection) | Up to 2 × 10^7^ cells per plate (6-well) of iPSCs within 24 days | NA | Hypoxia |
| Gutbier et al., 2020 | BMP4, VEGF, SCF, IL-3, M-CSF | | 14 days | 16 weeks  (8 weeks’ accumulation+8 weeks’ maintenance) | 1×10^4^ seeding iPSCs/EB | 20-60 monocytes/iPSC | 70-250 cells/iPSC | (1-3)×10^5^ macrophage progenitors per EB per week after day14 | pHrodo Zymosan | NA |
| Lopez-Yrigoyen et al.,2020 | BMP4, VEGF, SCF, IL-3, M-CSF | | 16 days | 3 months | Unfixed cell numbers/  EB | ＜5 monocytes /iPSC | ~7.8×10^7^/6 well plate | 2.59 × 10^6^ ± 0.54 cells  per plate (6-well) on days 16–28; 4.64 × 10^6^ ± 0.94 cells per plate (6-well) on day 28-80 | pHrodo Zymosan | NA |
| Monkley et al.,2020 | BMP4, VEGF, SCF, bFGF, TPO, FLT3L,  IL-3, GM-CSF, M-CSF | | 13-15  days | 18~39 days | 3×10^4^ seeding iPSCs/EB | ~10 monocytes /iPSC | 3.5 x10^7^ cells of a single differentiation | (2.5–4) x10^6^ CD14^+^ monocytes per harvest and up to 3.5 x10^7^ cells over the course of a single differentiation | pHrodo S. aureus | CD14^+^ sorting |
| Our data | bFGF, BMP4, VEGF, SCF, IL-3, M-CSF | | 14 days | 30 days | 8000 seeding iPSCs/EB | ~50 macrophages /iPSC | ~50 macrophages /iPSC | close to 50-fold of starting iPSCs, about ~2×10^7^ macrophages per plate (96-well) in a month | Latex beads;  Nalm6/Reh/  Raji cells | NA |
| **Monolayer culture protocols** | | | | | | | | | | |
| Senju et al., 2011 | | GM-CSF, M-CSF | 30 days | >35 days | / | NA | NA | NA | Zymosan; amyloid β; BALL-1 | NA |
| Yanagimachi et al.,2013 | | BMP4, VEGF, SCF, bFGF, TPO, FLT3L, IL-3, GM-CSF, M-CSF | 16-28 days | 24-36  days | / | (6-10)×10^6^ monocytes/30 colonies of iPSCs | (6-10)×10^6^ monocytes/30 colonies of iPSCs | （1.36±0.3）×10^6^ monocytes/30 clusters of iPSCs, 5–6 times | NA | CD14^+^ sorting |
| Takata et al., 2017 | | BMP4, VEGF, CHIR99021, SCF, bFGF, DDK1,  IL-3, IL-6, M-CSF | 16 days | 25 days | / | NA | 10-20 cells/iPSC | 10-20 cells per starting primary stem cell | fluorescent beads;  amyloid-β1-42 | Hypoxia |
| Cao et al., 2019 | | BMP4, Activin A, CHIR99021, SB431542, VEGF, SCF, bFGF, TPO, IL-3, IL-6, M-CSF | 15 days | 19 days | / | NA | 36.83±10.40 monocytes/  iPSC | 36.83 ± 10.40 monocytes/  hiPSC or 15.47 ± 4.37×10^6^  monocytes/  plate  (12-well) | pHrodo Green E. coli;  Jurkat cells | CD14^+^ sorting |
| Cui et al., 2021 | | BMP4, VEGF, CHIR99021, SB431542, bFGF, Tpo, IL-3, FLT3L, SCF, IL-6, GM-CSF, M-CSF | 22 days | NA | / | NA | 20000 monocytes/  iPSC | 1.8 × 10^6^  per single clone or 2 × 10^4^ per seeded hiPSC | pHrodo Red Escherichia coli | CD14^+^ sorting |
