# Supplementary material for: Optimizing the Method for Differentiation of Macrophages from Human Induced Pluripotent Stem Cells
Source: Stem Cells Int. 2022 Mar 3;2022:6593403. doi: 10.1155/2022/6593403 (PMC8913134; doi:10.1155/2022/6593403)
Supplement: Supplementary Materials — Figure S1: flow cytometric analysis of cells on day 14, day 22, and day 30. (a) The percentages of different markers (CD34+CD45+, CD45+CD14+, and CD45+CD11b+) of P3500 protocol (upper panel) and P8000 protocol (lower panel) on day 14. (b) The percentages of different markers (CD45+CD14+ and CD45+CD11b+) of P3500 protocol (upper panel) and P8000 protocol (lower panel) on day 22. (c) The percentages of different markers (CD45+CD14+ and CD45+CD11b+) of P3500 protocol (upper panel) and P8000 protocol (lower panel) on day 30. Figure S2: validation of the antitumor effect of macrophages in vivo. (a) A line chart of tumor volume over time and (b) the weight of tumors after the mice were sacrificed. (c) Hematoxylin-eosin staining of paraffin sections of tumor tissues. Arrows: macrophages. Scale bars, 250 μm in the left column, 50 μm in the middle and right column. Table S1: summary of protocols for differentiation of macrophages from iPSCs. A list of main publications for macrophage differentiation protocols including coculture with OP9 cells, EB-based protocols, and monolayer cultivation (tissue macrophage subsets such as microglia are not included). Movie: the phagocytosis of IPSDMs toward Reh-Hoechst 33342 was clearly observed in real-time fluorescence imaging of living cells (https://drive.google.com/file/d/1rLwt6xMU46n2u4J5JIOOqWWyHEeGCf7D/view?usp=sharing). [file 6593403.f1.zip › Supplemental Information.docx]

**Supplemental Information**

**Figure S1. Flow-cytometric analysis of cells on Day 14, Day 22, and Day 30.**

1. The percentages of different markers (CD34^+^CD45^+^, CD45^+^CD14^+^, and CD45^+^CD11b^+^) of P3500 protocol (upper panel) and P8000 protocol (lower panel) on Day 14.
2. The percentages of different markers (CD45^+^CD14^+^ and CD45^+^CD11b^+^) of P3500 protocol (upper panel) and P8000 protocol (lower panel) on Day 22.
3. The percentages of different markers (CD45^+^CD14^+^ and CD45^+^CD11b^+^) of P3500 protocol (upper panel) and P8000 protocol (lower panel) on Day 30.

**Figure S2. Validation of the anti-tumor effect of macrophages in vivo.**

(a) A line chart of tumor volume over time and (b) the weight of tumors after the mice were sacrificed.

(c) Hematoxylin-Eosin staining of paraffin sections of tumor tissues. Arrows: macrophages. Scale bars, 250µm in the left column, 50µm in the middle and right column.

**Table S1. Summary of protocols for differentiation of macrophages from iPSCs.**

A list of main publications for macrophage differentiation protocols including co-culture with OP9 cells, EB-based protocols, and monolayer cultivation. (Tissue macrophage subsets such as microglia are not included)

**Movie**

The phagocytosis of IPSDMs toward Reh-Hochest33342 was clearly observed in real-time fluorescence imaging of living cells.

<https://drive.google.com/file/d/1rLwt6xMU46n2u4J5JIOOqWWyHEeGCf7D/view?usp=sharing>

**Supplemental references**

Ackermann M, Kempf H, Hetzel M, et al. Bioreactor-based mass production of human iPSC-derived macrophages enables immunotherapies against bacterial airway infections [J]. Nature Communications, 2018, 9(1): 5088.

Brault J, Goutagny E, Telugu N, et al. Optimized Generation of Functional Neutrophils and Macrophages from Patient-Specific Induced Pluripotent Stem Cells: Ex Vivo Models of X(0)-Linked, AR22(0)- and AR47(0)- Chronic Granulomatous Diseases [J]. Biores Open Access, 2014, 3(6): 311-326.

Choi K D, Vodyanik M, Slukvin, II. Hematopoietic differentiation and production of mature myeloid cells from human pluripotent stem cells [J]. Nat Protoc, 2011, 6(3): 296-313.

Cao X, Yakala G K, van den Hil F E, et al. Differentiation and Functional Comparison of Monocytes and Macrophages from hiPSCs with Peripheral Blood Derivatives [J]. Stem Cell Reports, 2019, 12(6): 1282-1297.

Cui D, Franz A, Fillon S A, et al. High-Yield Human Induced Pluripotent Stem Cell-Derived Monocytes and Macrophages Are Functionally Comparable With Primary Cells [J]. Front Cell Dev Biol, 2021, 9: 656867.

Gutbier S, Wanke F, Dahm N, et al. Large-Scale Production of Human iPSC-Derived Macrophages for Drug Screening [J]. International Journal of Molecular Sciences, 2020, 21(13): 4808.

Kambal A, Mitchell G, Cary W, et al. Generation of HIV-1 resistant and functional macrophages from hematopoietic stem cell-derived induced pluripotent stem cells [J]. Mol Ther, 2011, 19(3): 584-593.

Lachmann N, Ackermann M, Frenzel E, et al. Large-Scale Hematopoietic Differentiation of Human Induced Pluripotent Stem Cells Provides Granulocytes or Macrophages for Cell Replacement Therapies [J]. Stem Cell Reports, 2015, 4(2): 282-296.

Lopez-Yrigoyen M, May A, Ventura T, et al. Production and Characterization of Human Macrophages from Pluripotent Stem Cells [J]. J Vis Exp, 2020, (158).

Monkley S, Krishnaswamy J K, Göransson M, et al. Optimised generation of iPSC-derived macrophages and dendritic cells that are functionally and transcriptionally similar to their primary counterparts [J]. PLoS One, 2020, 15(12): e0243807.

Mukherjee C, Hale C, Mukhopadhyay S. A Simple Multistep Protocol for Differentiating Human Induced Pluripotent Stem Cells into Functional Macrophages [J]. Methods Mol Biol, 2018, 1784: 13-28.

Senju S, Haruta M, Matsumura K, et al. Generation of dendritic cells and macrophages from human induced pluripotent stem cells aiming at cell therapy [J]. Gene Ther, 2011, 18(9): 874-883.

Takata K, Kozaki T, Lee C Z W, et al. Induced-Pluripotent-Stem-Cell-Derived Primitive Macrophages Provide a Platform for Modeling Tissue-Resident Macrophage Differentiation and Function [J]. Immunity, 2017, 47(1): 183-198.e6.

Shi J, Xue C, Liu W, et al. Differentiation of Human-Induced Pluripotent Stem Cells to Macrophages for Disease Modeling and Functional Genomics [J]. Curr Protoc Stem Cell Biol, 2019, 48(1): e74.

Wilgenburg B v, Browne C, Vowles J, et al. Efficient, Long Term Production of Monocyte-Derived Macrophages from Human Pluripotent Stem Cells under Partly-Defined and Fully-Defined Conditions [J]. PLOS ONE, 2013, 8(8): e71098.

Yanagimachi M D, Niwa A, Tanaka T, et al. Robust and Highly-Efficient Differentiation of Functional Monocytic Cells from Human Pluripotent Stem Cells under Serum- and Feeder Cell-Free Conditions [J]. PLOS ONE, 2013, 8(4): e59243.

Zhang H, Xue C, Shah R, et al. Functional analysis and transcriptomic profiling of iPSC-derived macrophages and their application in modeling Mendelian disease [J]. Circ Res, 2015, 117(1): 17-28.
