# Supplementary figures and images for: Optimizing the Method for Differentiation of Macrophages from Human Induced Pluripotent Stem Cells
Source: Stem Cells Int. 2022 Mar 3;2022:6593403. doi: 10.1155/2022/6593403 (PMC8913134; doi:10.1155/2022/6593403)

Figure S2

a

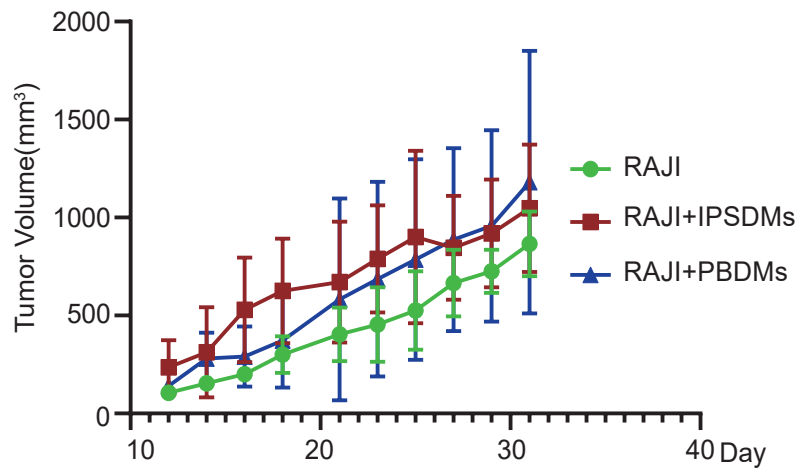

b

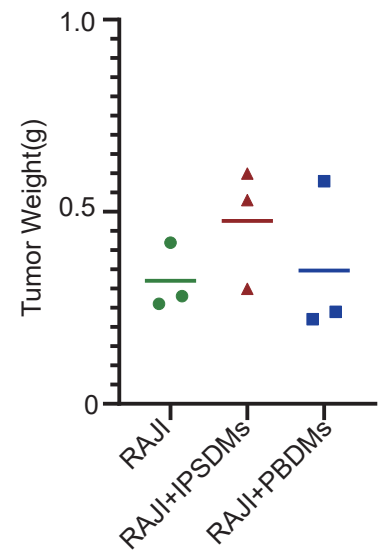

c

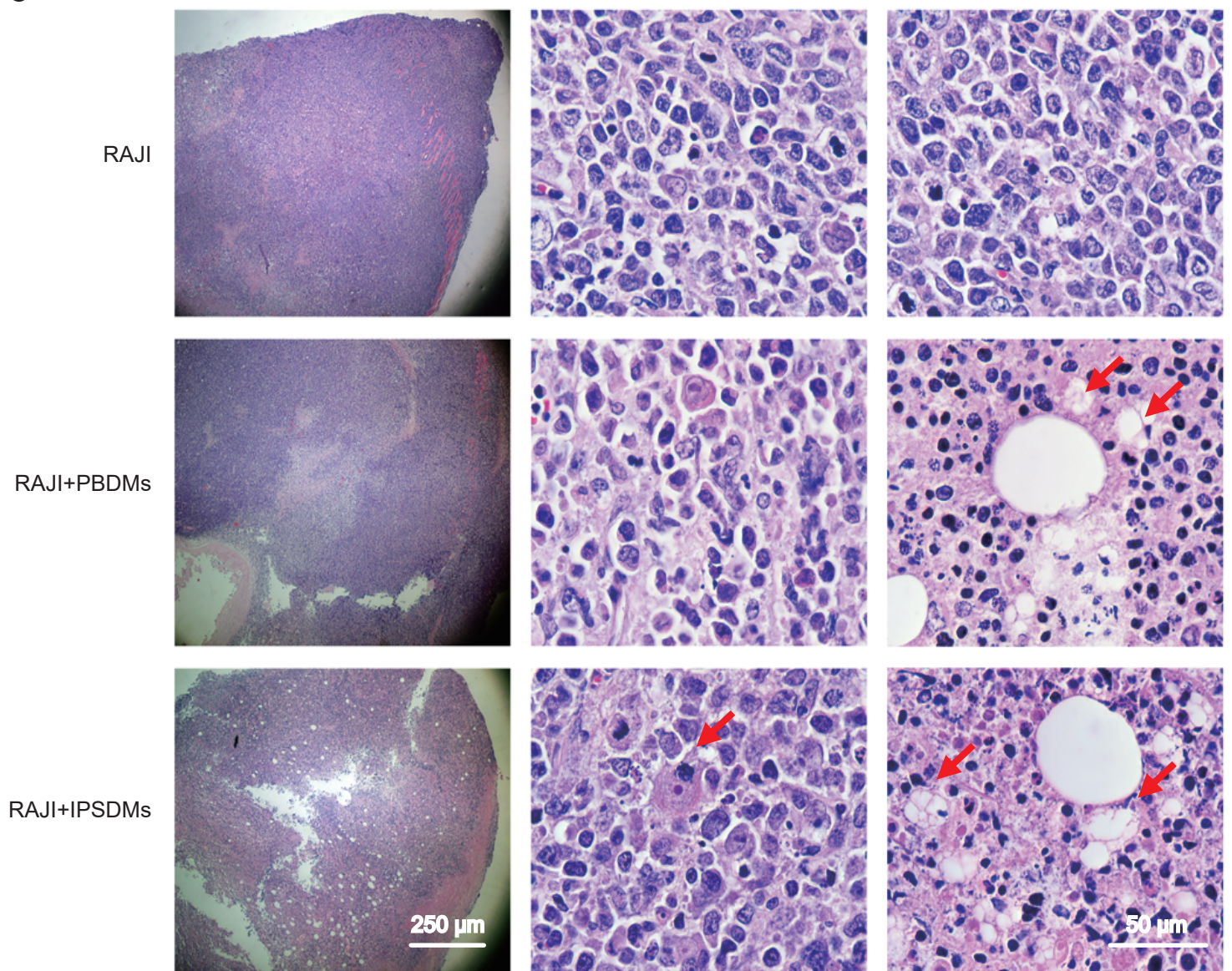

Supplement: Supplementary Materials — Figure S1: flow cytometric analysis of cells on day 14, day 22, and day 30. (a) The percentages of different markers (CD34+CD45+, CD45+CD14+, and CD45+CD11b+) of P3500 protocol (upper panel) and P8000 protocol (lower panel) on day 14. (b) The percentages of different markers (CD45+CD14+ and CD45+CD11b+) of P3500 protocol (upper panel) and P8000 protocol (lower panel) on day 22. (c) The percentages of different markers (CD45+CD14+ and CD45+CD11b+) of P3500 protocol (upper panel) and P8000 protocol (lower panel) on day 30. Figure S2: validation of the antitumor effect of macrophages in vivo. (a) A line chart of tumor volume over time and (b) the weight of tumors after the mice were sacrificed. (c) Hematoxylin-eosin staining of paraffin sections of tumor tissues. Arrows: macrophages. Scale bars, 250 μm in the left column, 50 μm in the middle and right column. Table S1: summary of protocols for differentiation of macrophages from iPSCs. A list of main publications for macrophage differentiation protocols including coculture with OP9 cells, EB-based protocols, and monolayer cultivation (tissue macrophage subsets such as microglia are not included). Movie: the phagocytosis of IPSDMs toward Reh-Hoechst 33342 was clearly observed in real-time fluorescence imaging of living cells (https://drive.google.com/file/d/1rLwt6xMU46n2u4J5JIOOqWWyHEeGCf7D/view?usp=sharing). [file 6593403.f1.zip › Figure S2.pdf]

Figure S1

a

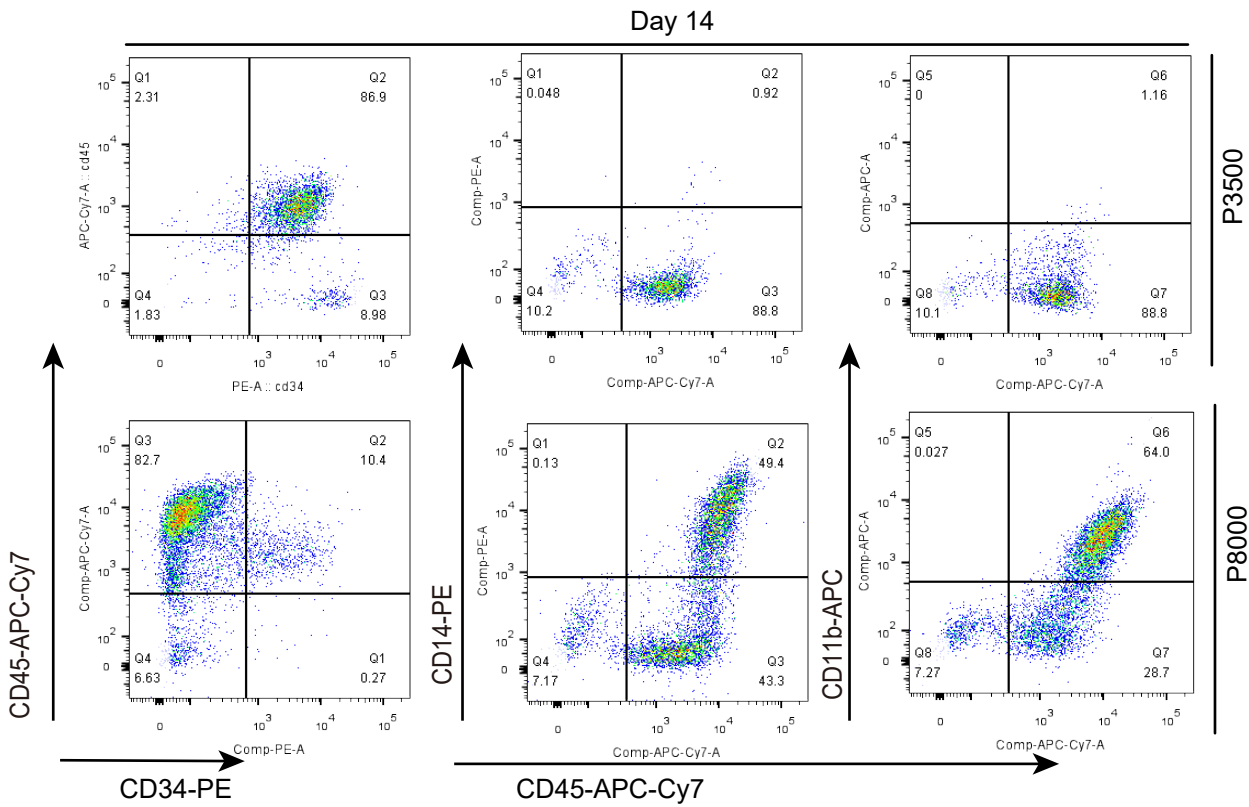

b

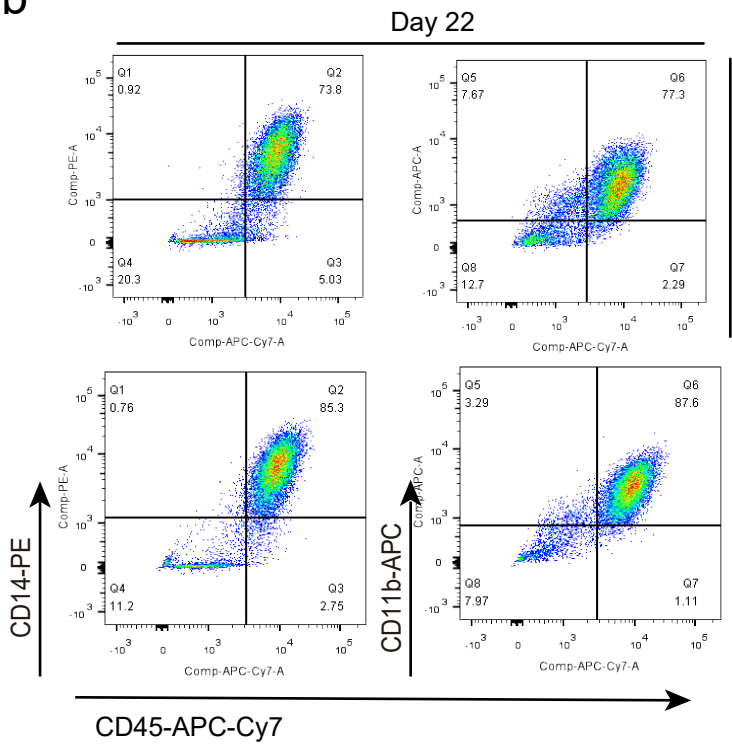

c

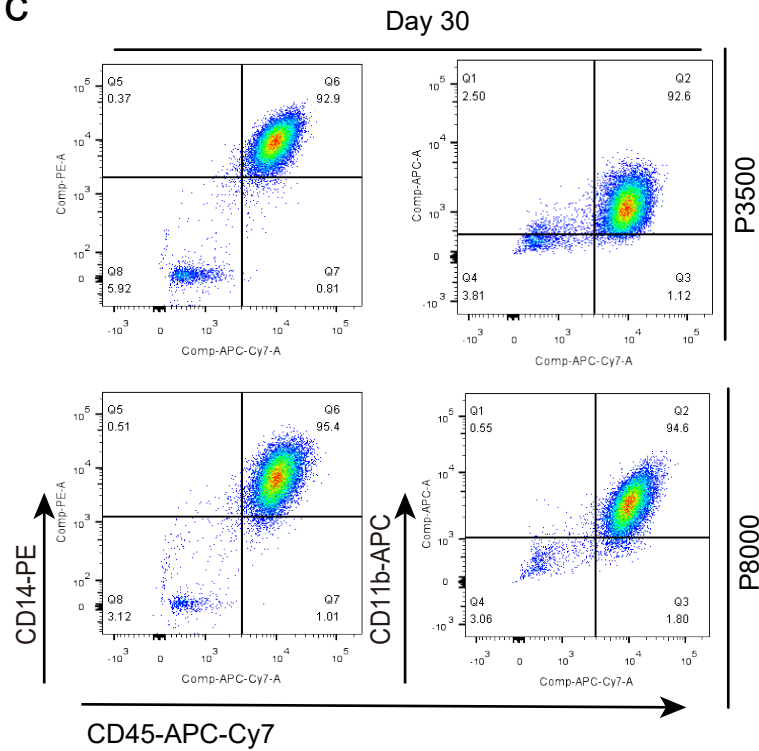

Supplement: Supplementary Materials — Figure S1: flow cytometric analysis of cells on day 14, day 22, and day 30. (a) The percentages of different markers (CD34+CD45+, CD45+CD14+, and CD45+CD11b+) of P3500 protocol (upper panel) and P8000 protocol (lower panel) on day 14. (b) The percentages of different markers (CD45+CD14+ and CD45+CD11b+) of P3500 protocol (upper panel) and P8000 protocol (lower panel) on day 22. (c) The percentages of different markers (CD45+CD14+ and CD45+CD11b+) of P3500 protocol (upper panel) and P8000 protocol (lower panel) on day 30. Figure S2: validation of the antitumor effect of macrophages in vivo. (a) A line chart of tumor volume over time and (b) the weight of tumors after the mice were sacrificed. (c) Hematoxylin-eosin staining of paraffin sections of tumor tissues. Arrows: macrophages. Scale bars, 250 μm in the left column, 50 μm in the middle and right column. Table S1: summary of protocols for differentiation of macrophages from iPSCs. A list of main publications for macrophage differentiation protocols including coculture with OP9 cells, EB-based protocols, and monolayer cultivation (tissue macrophage subsets such as microglia are not included). Movie: the phagocytosis of IPSDMs toward Reh-Hoechst 33342 was clearly observed in real-time fluorescence imaging of living cells (https://drive.google.com/file/d/1rLwt6xMU46n2u4J5JIOOqWWyHEeGCf7D/view?usp=sharing). [file 6593403.f1.zip › Figure S1 (1).pdf]
